# Simultaneous Dry and Gel-Based High-Density Electroencephalography Recordings

**DOI:** 10.3390/s23249745

**Published:** 2023-12-11

**Authors:** Patrique Fiedler, Uwe Graichen, Ellen Zimmer, Jens Haueisen

**Affiliations:** 1 Institute of Biomedical Engineering and Informatics, Technische Universität Ilmenau, 98693 Ilmenau, Germany; 2 Department of Biostatistics and Data Science, Karl Landsteiner University of Health Sciences, 3500 Krems an der Donau, Austria

**Keywords:** electroencephalography, EEG, dry electrodes, validation, brain imaging, spatial frequencies, spatial harmonics

## Abstract

Evaluations of new dry, high-density EEG caps have only been performed so far with serial measurements and not with simultaneous (parallel) measurements. For a first comparison of gel-based and dry electrode performance in simultaneous high-density EEG measurements, we developed a new EEG cap comprising 64 gel-based and 64 dry electrodes and performed simultaneous measurements on ten volunteers. We analyzed electrode–skin impedances, resting state EEG, triggered eye blinks, and visual evoked potentials (VEPs). To overcome the issue of different electrode positions in the comparison of simultaneous measurements, we performed spatial frequency analysis of the simultaneously measured EEGs using spatial harmonic analysis (SPHARA). The impedances were 516 ± 429 kOhm (mean ± std) for the dry electrodes and 14 ± 8 kOhm for the gel-based electrodes. For the dry EEG electrodes, we obtained a channel reliability of 77%. We observed no differences between dry and gel-based recordings for the alpha peak frequency and the alpha power amplitude, as well as for the VEP peak amplitudes and latencies. For the VEP, the RMSD and the correlation coefficient between the gel-based and dry recordings were 1.7 ± 0.7 μV and 0.97 ± 0.03, respectively. We observed no differences in the cumulative power distributions of the spatial frequency components for the N75 and P100 VEP peaks. The differences for the N145 VEP peak were attributed to the different noise characteristics of gel-based and dry recordings. In conclusion, we provide evidence for the equivalence of simultaneous dry and gel-based high-density EEG measurements.

## 1. Introduction

Electroencephalography (EEG) has been widely used in clinical practice and medical research to measure electrical brain activity. High-density EEG (HD-EEG) typically comprises 64 or more channels arranged in a cap. The gold standard for EEG sensors are gel-based Ag/AgCl electrodes, where an electrolyte gel or paste is placed between the electrode and the scalp for optimal contact. 

In recent years, dry electrodes were developed that contact the scalp directly without an electrolyte gel or paste [1,2,3,4,5,6,7,8]. Different types of electrode shapes (e.g., needles, pins, and spiders) and materials have been proposed [9,10,11,12] for both completely dry and semi-dry [13,14,15,16,17] electrode concepts. Dry electrodes have several advantages over gel-based electrodes. For example, EEG caps with dry electrodes can be self-applied and easily used in settings outside a laboratory or clinic [2,18,19]. Their preparation and cleaning times are considerably shorter, especially for higher numbers of channels [2,18]. However, currently, channel dropout rates are higher for dry electrode recordings [2,18], and they are more prone to movement artifacts [12]. Thus, it is important to test the performance of the dry electrodes in direct comparison with the gold standard electrodes. In addition to mechanical, electrical, and chemical characterizations of both types of electrodes [10,15,19], comparisons in studies with human volunteers are important to assess electrode performance under realistic conditions and for distinct applications like laboratory settings in research [1,2,3,4,5,11,12,13,18] and clinical diagnostics [6], emergency applications [20,21], brain–computer interfacing [22,23], home settings [16], or mobile recordings in, for example, sports and movement sciences [24,25]. Each of these applications poses specific requirements for the sensors and sensor applicators, but across all of them, ensuring objective comparison metrics, reproducibility, and minimizing the impact of not sensor-related influences and parameters are key aspects during study design. 

There are two principal ways to compare the performance of new electrode systems in practical applications: *serial* or *simultaneous* measurements [26]. In *serial* measurements, the volunteer is first measured with one type and then with the second type of electrodes, most commonly in a randomized order to avoid systematic errors. This paradigm is also referred to as the *same place–different time* comparison. The advantage of serial measurements is that more or fewer identical positions can be used for both types of electrodes. However, the recorded brain activity is different due to its intra- and inter-individual variability over time. Consequently, data comparison is usually focused on interfacial impedance, channel reliability, and spectral characteristics, while time-domain analyses can only be performed for deterministic components, like evoked activity. *Simultaneous* (or *parallel* and *concurrent*) measurements, where the cap comprises a set of both types of electrodes, are also referred to as *same time–different place* measurements. In the context of the paper at hand, we will refer to this setup as a *simultaneous* measurement. Several papers have demonstrated the utility of simultaneous measurements [3,7,11,22,27,28,29,30,31,32,33], specifically the advantage that the same type of brain activity is measured but the electrodes are in different positions. Typically, adjacent electrode positions are chosen for comparing the results between the two types of electrodes. In contrast to sequential measurements, the recorded spontaneous EEG data of adjacent electrodes can also be compared in the time domain, although differences due to unavoidable different electrode positions must be taken into consideration during the interpretation of the results.

For a low number of channels (i.e., low-density EEG), both serial [7,10,12,34,35,36] and simultaneous [3,7,11,22,27,28,29,30,31,32] measurements of dry and gel-based electrodes have been reported. However, only sequential measurements have been reported for the comparison of gel-based and dry high-density EEGs [1,2]. 

With the aim of the first comparison of gel-based and dry electrode performance in simultaneous high-density EEG measurements, we introduced a new EEG cap with 64 gel-based and 64 dry electrodes. We performed simultaneous measurements on ten volunteers using an established validation paradigm comprising both spontaneous and evoked EEG activities. We propose an approach to overcome the issue of different electrode positions in the comparison of simultaneous measurements by applying spatial harmonic analysis (SPHARA) [37,38]. We provide a spatial frequency analysis of our simultaneously measured EEGs.

## 2. Materials and Methods

### 2.1. EEG Sensors

**Figure 1:**
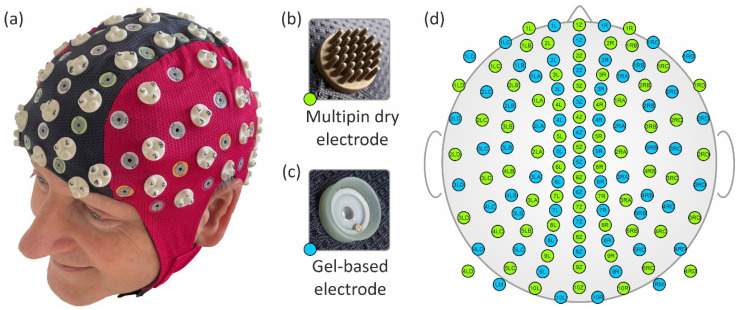
Photos of the assembled EEG cap on a volunteer (**a**), the dry multipin electrode (**b**), the commercial gel-based electrode (**c**), and the equidistant layout of the 64 gel-based (blue) and 64 dry (green) EEG channels (**d**).

We integrated 64 gel-based and 64 dry EEG electrodes into a common cap. The gel-based electrodes were sintered Ag/AgCl electrodes taken from a commercial cap (waveguard™ original, ANT Neuro BV, Hengelo, The Netherlands). The dry electrodes were similar to our previous dry electrodes [1,39] and comprised 30 pins on a common base plate. The dry electrode substrate material was thermoset polyurethane (Biresin U1419, Sika Chemie GmbH, Bad Urach, Germany) with a Shore A hardness of 98. The substrates were coated with Ag/AgCl in a multi-phase chemical process [1,40], ensuring the formation of a highly conductive layer on the non-conductive polymer substrate. The substrate, the coating material, and their combination have passed their mechanical durability [2] and biocompatibility tests for their use on healthy skin. Coaxial cables were directly soldered to the back of the electrodes, supporting the use of active shielding for reduced susceptibility to environmental noise. The proprietary active shielding implementation was integrated into the used EEG amplifier model, applying a defined potential to the outer shield of the coaxial cable. Using identical amplifiers and cabling for both electrode types ensured equivalent recording conditions for both compared datasets. See Figure 1 for a photo of both types of electrodes.

### 2.2. EEG Cap

We used the fabric and the coaxial cabling of a commercial double-layer EEG cap (waveguard™ original, ANT Neuro BV, Hengelo, The Netherlands) and integrated the gel-based and dry electrodes. The cabling was completely covered between the two fabric layers, avoiding exposure to mechanical stress during the application and removal of the cap. The dry electrodes had a total height of 9 mm (6 mm between the head and the fabric), while the gel-based electrodes had a total height of 6 mm (approx. 5 mm between the head and the fabric). If the electrodes are positioned too close to each other, individual gel-based electrodes may lift off. This can lead to gel leakage and conductive bridges. Consequently, sufficient spacing between electrodes is needed. We tested this in a separate cap and found the minimum electrode spacing to be approx. 20 mm. Other requirements for the construction of the cap were approximately alternating gel-based and dry electrode positions; approximately similar local and global distribution for dry and gel-based electrode sets; approximately covering the same overall head area; an equidistant layout; and common ground and reference positions for both electrode sets. 

Based on a standard 128-channel equidistant layout [33], we manually distributed the gel-based and dry electrodes and adjusted their positions as required to meet the aforementioned constraints. The resulting mean inter-electrode distance between adjacent sensors, considering both sensor types together, was 29 ± 6 mm (mean ± standard deviation). Considering the sensor types separately, the mean inter-electrode distance was 37 ± 7 mm for gel-based electrodes and 40 ± 8 mm for dry electrodes. A photo and the final electrode layout of the cap are depicted in Figure 1a and Figure 1d, respectively. Please note that each of the two electrode-specific subsets of the overall montage comprises the same electrode number and similar distribution, enabling direct comparison of adjacent positions, as indicated by corresponding channel labels.

### 2.3. In Vivo Measurements

Ten healthy volunteers (four females) participated in this study (age: 29.5 ± 7.0 years). The average head circumference was 57.7 ± 1.3 cm, and the distance from nasion to inion was 36.2 ± 1.5 cm, allowing for the use of one medium-sized cap for all volunteers. The hair length was approx. 21 ± 20 cm. The inclusion criteria for volunteers were: a healthy neurological, psychological, and dermatological state, no history of drug abuse, and a minimum of 7 h of sleep the night before study participation. Consequently, exclusion criteria included medication for neurological, psychological, or dermatological pathologies, as well as the existence of skin lesions on the head. Moreover, to minimize the highly variable influence of sweat, fat layers, and styling products on the electrode–skin interface, the volunteers were instructed to wash their hair using pH-neutral shampoo on the morning of the day of their study participation. This study was approved by the local Ethics Committee of the University Hospital Jena, Jena, Germany, and complied with the ethical standards outlined in the Declaration of Helsinki. Prior to their participation, all volunteers were informed about the study purpose, procedures, and involved equipment and materials. The volunteers had the opportunity to clarify any open questions and provided written informed consent before their participation in the study.

After placing the cap, the gel-based electrodes were filled with electrolyte gel (Electro-Gel, Electro-Cap International Inc., Eaton, OH, USA). Self-adhesive pre-gelled hydrogel ground and reference electrodes (Kendall ECG electrodes H124SG, Covidien LLC, Mansfield, MA, USA) were placed at the left and right mastoids. The two electrode sets of the cap were connected to two identical commercial 64-channel referential EEG amplifiers (eego™ amplifier EE-225, ANT Neuro BV, Hengelo, The Netherlands) in a cascaded HD-EEG setup. The amplifiers provide an input impedance of >1 GOhm, a common mode rejection ratio of >100 dB and support active shielding. A sampling rate of 2048 samples/s was used. Both amplifiers recorded data from one of the electrode sets (gel-based and dry, respectively) only sharing common reference and ground electrodes, thus ensuring independent data acquisition. All electrode–skin impedance measurements were performed using the integrated measurement functions of the amplifier. Data synchronization between both setups was ensured using the eego™ control software cascading functions (Version 1.8.2, ANT Neuro BV, Hengelo, The Netherlands) and by the triggers of the stimulation device transmitted to both amplifiers. 

The measurement paradigm consisted of eyes in open and closed resting states (three minutes each), triggered eye blinks, and pattern reversal visual evoked potentials (VEPs). Indications for eye blinks, as well as pattern reversal stimulation for the VEPs, were presented using eevoke software (eemagine Medical Imaging Solutions GmbH, Berlin, Germany). All recordings were performed in a relaxed, sitting position.

### 2.4. SPHARA

The use of different sensor types in a combined EEG setup allows for capturing the electric potential of the same generating brain sources simultaneously with both electrode types. Due to the physical space requirement of the electrodes, the electric potential cannot be recorded at identical electrode positions (i.e., spatial sampling points) on the head surface using two types of electrodes. To address this issue, we applied spatial harmonic analysis (SPHARA) [38,39] to compare the measured signals from both types of electrodes.

SPHARA enables spatial Fourier analysis for data obtained using multi-sensor systems, consisting of sensors placed at irregularly arranged positions on an arbitrarily shaped surface, such as the EEG sensor setup (see Figure 1a,c). The SPHARA approach can be interpreted as a generalization of the spatial Fourier analysis for arbitrary surfaces. The sensor positions can be considered as vertices in vector space ($R^{3})$, and can be used to specify a triangular mesh (M=V,E,T) with sets of vertices (*V*), edges (*E*), and triangles (*T*). For a function defined on the vertices of the triangular mesh f: V→R, a discrete Laplace–Beltrami operator (Δ) in matrix notation can be defined as $\Delta \vec{f}=-L\vec{f}$. Since we aimed to use SPHARA for a quantitative analysis, we chose a geometric approach for the discretization of the Laplace–Beltrami operator, the FEM approach $L=B^{-1}Q$, with the mass matrix (*B*) and the stiffness matrix (*Q*). Thereafter, the SPHARA basis $\Psi =(|\vec{\psi_{1}}|\vec{\psi_{2}}|\ldots |\vec{\psi_{n}}|)$ was determined by solving the generalized Laplacian eigenvalue problem, $Q\vec{\psi_{i}}=\lambda_{i}B\vec{\psi_{i}}$, with the eigenvalues $\left(\lambda_{i}\right)$ and the eigenvectors ($\vec{\psi_{i}}$), i.e., the eigenanalysis of this discrete Laplace–Beltrami operator. The recorded multi-sensor data were transformed to the space of spatial frequencies by projections into the space spanned by the SPHARA basis functions (Ψ).

The spatial frequencies of the SPHARA basis functions were determined by the shape and area of the head covered by the electrodes. The spatial signal power detected with the multi-sensor systems was also determined by the area covered. During an EEG measurement, both electrode types were positioned on the same head shape. Thus, the triangular grids created independently for both electrode types have essentially the same shape. Both electrode types were alternately placed on the edge of our EEG cap so that the edges of the two triangular grids were highly similar. It follows that the shape and covered area of the two triangular grids were very similar and, thus, the SPHARA bases used for the spatial Fourier analysis exhibited highly comparable spatial–spectral properties (see Figure 1).

The discrete data obtained with a multi-channel system consisting of *n* electrodes were specified in the space $R_{X}^{n}$ on a canonical basis. In this space, differences in terms of least squares were quantified using an $L^{2}$-based metric, $L^{2}\left(R^{n}\right)$. The Fourier transform and thus also the SPHARA transform is an isometry of $L^{2}\left(R^{n}\right)$; the following holds true ${\left\|f\right\|}_{L^{2}\left(R_{X}^{n}\right)}={\left\|\hat{f}\right\|}_{L^{2}\left(R_{SPHARA}^{n}\right)}$ with the *n* dimensional space, $R_{SPHARA}^{n},$ spanned by the SPHARA basis (Ψ). It follows that differences in the sense of least squares can be determined equally in both the canonical spatial space and the space spanned by the SPHARA basis.

### 2.5. Analysis and Statistics

The measured electrode–skin impedances were averaged after applying a threshold of 1 MOhm, in accordance with the manufacturer’s specifications for the EEG amplifier. The impedance of one gel-based electrode in a single volunteer was above this threshold and excluded from the analysis. The measured EEG data were visually inspected and disturbed channels and epochs were excluded from further evaluation. The EEG data were filtered with a 24 dB Butterworth bandpass filter (1–40 Hz) and a 36 dB Butterworth bandstop filter (48–52 Hz) to further reduce any remaining powerline interferences. The data were analyzed using custom MATLAB scripts (The Mathworks, Natick, MA, USA).

Using the Welch estimation method, we computed the power spectral density (PSD) for 30 s of eyes-closed resting-state EEG data for both gel-based and dry recordings. After filtering the raw VEP data, artifact-contaminated trials were manually identified and excluded before averaging the remaining trials. We calculated two independent estimations of the signal-to-noise ratio: SNR_max_ and SNR_MGFP_ [2]. The estimation of SNR_max_ was calculated for each volunteer’s individual channel with maximum N75 and P100 peak amplitudes. The signal was defined by the respective N75 and P100 peak amplitude values, while the noise was defined as the mean of the respective channel amplitude in a 50 ms baseline interval prior to stimulation, t = [−100, −50] ms. Subsequently, the mean global field power (MGFP) over all channels was calculated for an interval of 500 ms (1024 samples). The root-mean-square deviation (RMSD) and Pearson correlation coefficient of the MGFP were used to quantitatively evaluate the comparison between wet and dry electrodes in the VEP data. The SNR_MGFP_ was calculated for the respective N75 and P100 peak powers and the [−100, −50] ms noise interval of the MGFP.

## 3. Results

### 3.1. Impedances and Channel Reliability

**Figure 2:**
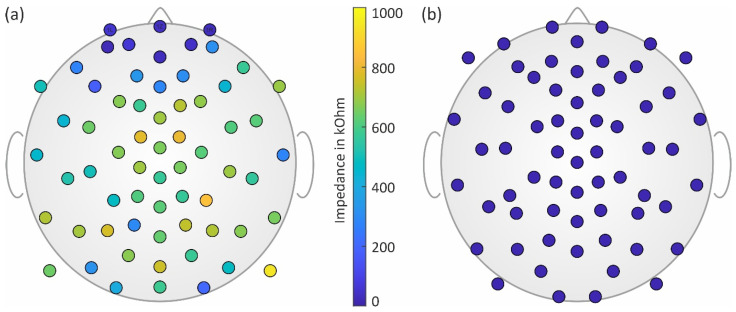
Topographic distribution of the electrode–skin impedances of (**a**) the dry electrodes and (**b**) the gel-based electrodes shown at their respective individual location subsets.

The mean electrode–skin impedance (mean ± std) of the dry electrodes calculated over all measurements was 516 ± 429 kOhm, while the mean impedance of the gel-based electrodes was 14 ± 8 kOhm. The topographic distribution of the impedances for both electrode types is shown in Figure 2.

In line with previous investigations [2], we found lower mean and standard deviation values of the dry electrodes’ impedance for frontal regions and higher values for central regions of the head. For all gel-based recordings, only one channel had to be excluded based on visual inspection. For dry recordings, on average, 77% of the channels were used for further analysis (range: 69–91%). Channel reliability of the dry electrodes was at its highest in the frontal regions and lowest in the central regions of the head. 

Based on the topographies and the measured impedance values, we were able to ensure that no conductive bridges existed between adjacent gel-based and dry electrodes, which could have been caused, e.g., by gel running.

### 3.2. Resting State EEG

**Figure 3:**
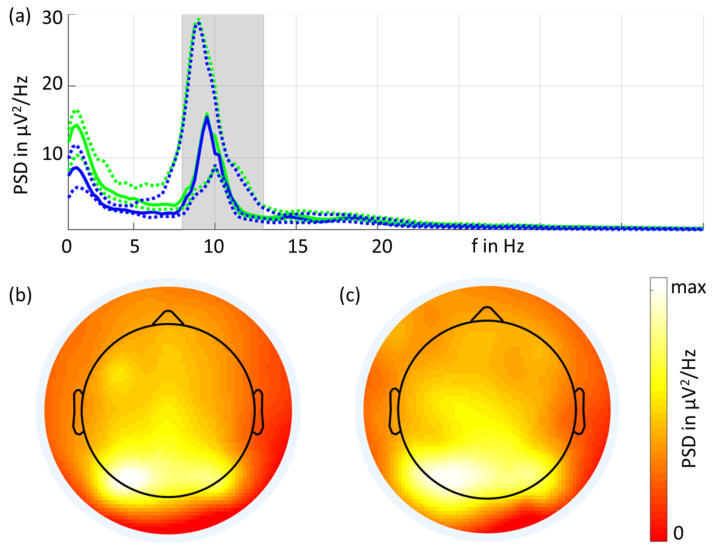
Power spectral density (**a**) in the frequency range from 1 Hz to 40 Hz for the gel-based (blue) and dry (green) electrodes (solid lines: median; dotted lines: 25% and 75% quartiles). The grand average 2D interpolated topographic plots for the gel-based (**b**) and dry (**c**) electrodes show the alpha band power (shaded band area in (**a**)).

The grand average of the eyes-closed resting-state EEG PSDs is shown in Figure 3. Overall, the spectral characteristics of the two types of electrodes were similar. Increased power in the alpha band was visible, and the largest peak was observed at 9.5 Hz for both types of electrodes. The PSD (mean ± std) at 9.5 Hz was 26 ± 28 μV^2^/Hz for the gel-based electrodes and 31 ± 38 μV^2^/Hz for the dry electrodes. The dry electrodes showed increased PSD values for frequencies below 8 Hz compared to the gel-based signals, which is in line with previous investigations [1,2]. 

Figure 3 also shows the grand average 2D topographic plots of the alpha band power in the eyes-closed condition for gel-based and dry recordings. The highest power was visible in the parietal and occipital channels, and the spatial distribution was very similar for both recordings.

### 3.3. Triggered Eye Blinks

**Figure 4:**
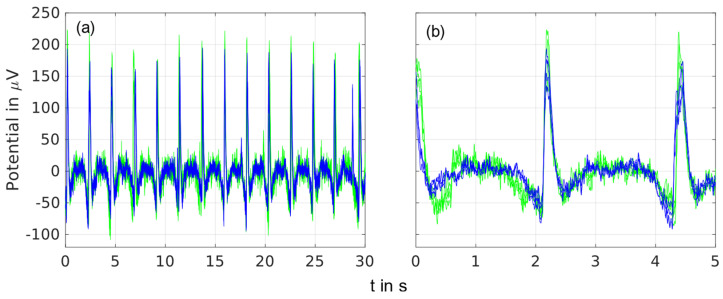
Overlay plot of spontaneous EEG recordings for the gel-based (blue) and dry (green) electrodes with triggered eye blinks of an exemplary volunteer recorded by the frontal electrodes at the 1L, 1LC, 1R, and 1RC positions and filtered by 1–40 Hz: (**a**) overlay of 50 s, and (**b**) overlay of 5 s.

Time domain overlay plots of spontaneous EEG recordings containing triggered eye blinks are shown in Figure 4 for recordings of 5 s and 30 s and four frontal electrodes. 

A similar signal shape was visible for both types of electrodes in the detailed 5-s recording as well as in the 30-s long-time recording. The baseline signals between the eye blinks were similar for the dry and gel-based electrodes.

### 3.4. Visual Evoked Potentials

**Figure 5:**
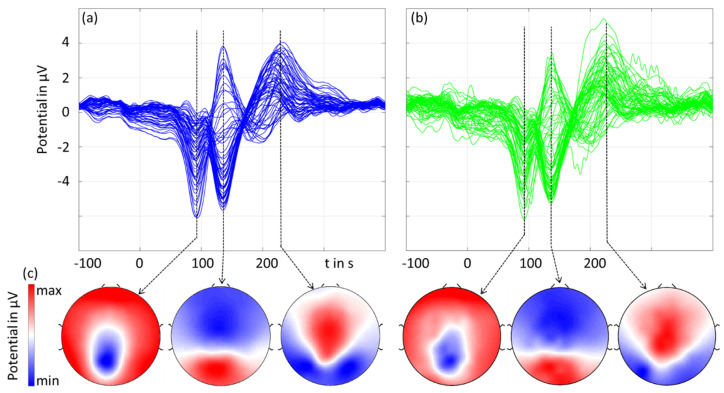
The grand average of the pattern reversal visual evoked potential: (**a**,**b**) butterfly plots of gel-based (blue) and dry (green) electrodes, and (**c**) 2D interpolated topographic mapping of the three main peaks of the VEP (normalized to the respective maximum amplitude).

Figure 5 shows the grand averages of the checkerboard pattern reversal VEP’s amplitudes and latencies. For the three main components at N75, P100, and N145, we plotted the spatial topographies accordingly with a respectively normalized colormap scale.

The results in the time domain (amplitude and latency) as well as the spatial domain were similar for the gel-based and dry electrodes. The dry recordings exhibited higher noise compared to the gel-based recordings. The latencies measured in the MGFP did not differ by more than 1 ms for the two types of recordings for all three peaks. The amplitudes (mean ± std) of the three peaks measured in the MGPF were 14.8 ± 4.1 μV (gel-based) and 13.8 ± 3.0 μV (dry), 25.8 ± 7.6 μV (gel-based) and 23.5 ± 6.2 μV (dry), and 14.2 ± 6.8 μV (gel-based) and 13.3 ± 4.9 μV (dry), respectively. The RMSD and the correlation coefficient between the gel-based and dry recordings calculated over all individual volunteers and channels were 1.7 ± 0.7 μV and 0.97 ± 0.03, respectively.

The SNR_max_ values of the P100 components of the VEP were 8.6 ± 7.2 for the dry electrodes and 8.9 ± 6.8 for the wet electrodes, respectively. For the N75 components, the SNR_max_ was 3.7 ± 2.2 and 4.8 ± 3.9, respectively. Moreover, the SNR_MGFP_ values were 4.3 ± 1.6 (dry, P100), 5.5 ± 1.7 (wet, P100), 2.7 ± 0.9 (dry, N75), and 3.4 ± 1.5 (wet, N75). 

### 3.5. Spatial Frequencies

**Figure 6:**
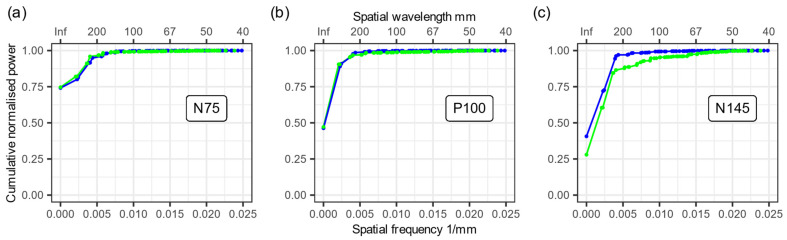
SPHARA analysis of the three main VEP peaks. Plots of the normalized cumulative power contributions of the spatial frequency components determined via SPHARA decomposition for the three main VEP peaks. (**a**) N75, (**b**) P100, and (**c**) N145 are displayed, recorded with gel-based (blue) and dry (green) electrodes.

We used the grand average data and performed a SPHARA decomposition of the three main VEP peaks separately for both types of electrodes. Based on the coefficients of this SPHARA decomposition, we determined a normalized cumulative power distribution of the spatial frequencies (see Figure 6). In the two plots for the VEP peaks N75 and P100, we verified an extremely high agreement between the normalized cumulative power distributions (see Figure 6a,b). However, a difference was observed between the gel-based and dry electrodes for the VEP peak N145 (see Figure 6c). This difference was caused by increased noise in individual dry electrode channels (see also Figure 5b). The noise in a few channels causes a higher energy contribution from spatially higher frequency components, and this leads to the observed difference in Figure 6c.

## 4. Discussion

We developed a novel high-density EEG cap for simultaneous dry and gel-based recordings and successfully performed measurements on ten volunteers. We found similar EEG signal quality for both electrode types in the time, frequency, and space domains. For the first time, these findings support the validation of our dry EEG electrodes in a simultaneous high-density multichannel measurement setup. Our results are in line with previous publications regarding low-density dry multichannel EEG and prove the functionality and applicability of the novel technology for HD-EEG. 

The new cap allows for simultaneous recordings of EEGs with different types of electrodes. By design, during simultaneous measurements, the spatial sampling positions of the different types of electrodes on the head surface are not in identical locations. The SPHARA-based approach allows for the comparison of EEG potential distributions recorded with different types of electrodes in a single cap. This approach exploits the fact that the frequencies in the discrete spatial spectra are essentially determined by the surface area covered by the sensor setup and by the distance between the sensors. We decomposed the spatial EEG potential distribution using the SPHARA approach and compared the energy contributions of the spatial frequency components. The obtained cumulative power contributions of the spatial frequency components support the validation of the new dry electrode concepts. The spatial frequency analysis of the simultaneously measured EEGs provides a direct means of comparing different electrode systems. Future studies may investigate further applications of SPHARA, e.g., reducing the impact of bad channels by applying spatial filtering and denoising [37]. 

Kutafina et al. [22] performed a comparison of a gel-based, medical-grade EEG system (21 electrodes) with a dry consumer-grade EEG system (14 electrodes) originally meant for BCI applications. In their study, both systems were applied simultaneously, and their data were recorded simultaneously with the aim to validate the recordings of the consumer-grade EEG system. They found a correlation coefficient of 0.64 between the gel-based and dry recordings. Different from our approach, the comparison by Kutafina et al. was for two completely independent systems, i.e., different amplifier models, different electrode holding mechanisms, different sampling frequencies, and different hardware filters [22]. In our study, only the electrodes were different, while all other components in the recording and analysis pipeline were the same for both the gel-based and dry electrode data. The differences in the setups of Kutafina et al. might partly explain the differences in the correlation between their gel-based and dry recordings. In our study at hand, no considerable differences were evident for spontaneous EEG recordings with both open eyes and closed eyes, triggered eyeblink artifacts, or evoked activity in each of the time, frequency, and spatial domain comparisons. We were able to provide quantitative evidence for the electrode’s equivalence of signal characteristics in the time domain using the RMSD and the Pearson correlation coefficient of the MGFP, as well as the SNR_max_ and SNR_MGFP_. Across all comparison domains and metrics, minor differences in the signal characteristics are in line with previous publications [1,2,18,24] and at the order of intra-individual variability [4,10,29,33]. 

Comfort is an important aspect of dry electrodes and has been reported to be limited for many state-of-the-art electrode concepts. Spring-loaded pins [6,28], special shapes [4,5,7], and flexible materials [8,11,39] have been proposed as eventual solutions. We investigated the comfort of our multipin dry electrodes under different application parameters, varying adduction force, and flexibility [39]. Moreover, we reported comfort for different layouts ranging from 32 to 256 channels [1,2,18,24,41] and in a multi-center study [18]. In these previous studies, the volunteers evaluated comfort on a scale from one (very comfortable) to ten (very painful). The reported average value comfort across these studies was in the range from two to four for a wearing time between 40 min and 60 min. 

The channel reliability in our study was at its lowest for the central region of the head, which was mainly due to the cut of the textile caps. Also, the impedances of the dry electrodes were at their highest in the central region. However, there was no direct correlation on a single-channel basis between impedance and channel reliability, which is in line with previous investigations [2,42]. For the used state-of-the-art EEG amplifiers with high input impedance and active shielding, impedances up to 800 kOhm showed no high levels of correlation with bad (including noisy) channels, according to previous studies [2]. We noted that both the order of magnitude of the grand average channel reliability as well as the observed regions of reduced channel reliability are in line with previous validation studies using sequential measurement setups and separate caps [1,2,18,24]. Moreover, similar topographies of reliability and impedance distributions have been observed for both low-density [40] and high-density dry EEG studies [1,2]. This observation supports the conclusion that the specific designs and different heights of the two electrode sets integrated into one cap did not result in considerable cross-sensor effects, i.e., the adduction of the dry electrodes was not considerably reduced due to adjacent gel-based electrodes. 

## 5. Conclusions

In conclusion, our study provides further evidence for the equivalence of the dry and gel-based EEG measurements, here for the first time with simultaneous high-density EEG measurements. It thus contributes to the validation efforts for new dry electrode concepts. These electrodes can be self-applied and have reduced preparation times as well as reduced cleaning effort. These advantages may open up new fields of application for high-density EEG recordings. Future studies may use the hybrid gel-based and dry EEG caps for investigations of their applicability in further fields of use and conditions, including, e.g., body movement. Moreover, we applied the SPHARA algorithm for spatial analysis and demonstrated its potential to be used for directly comparing the different electrode systems in addition to the different electrode positions. 

## Data Availability

All relevant data will be available from the corresponding authors upon reasonable request.
